# Robust increase in extreme summer rainfall intensity during the past four decades observed in China

**DOI:** 10.1038/srep38506

**Published:** 2016-12-05

**Authors:** Chan Xiao, Peili Wu, Lixia Zhang, Lianchun Song

**Affiliations:** 1 Beijing Climate Centre, China Meteorological Administration, Beijing, China; 2 Met Office Hadley Centre, Exeter, UK; 3 LASG, Institute of Atmospheric Physics, Chinese Academy of Sciences, Beijing, China; 4 Collaborative Innovation Centre on Forecast and Evaluation of Meteorological Disasters, Nanjing University of Information Science & Technology, Nanjing, China

## Abstract

Global warming increases the moisture holding capacity of the atmosphere and consequently the potential risks of extreme rainfall. Here we show that maximum hourly summer rainfall intensity has increased by about 11.2% on average, using continuous hourly gauge records for 1971–2013 from 721 weather stations in China. The corresponding event accumulated precipitation has on average increased by more than 10% aided by a small positive trend in events duration. Linear regression of the 95^th^ percentile daily precipitation intensity with daily mean surface air temperature shows a negative scaling of −9.6%/K, in contrast to a positive scaling of 10.6%/K for hourly data. This is made up of a positive scaling below the summer mean temperature and a negative scaling above. Using seasonal means instead of daily means, we find a consistent scaling rate for the region of 6.7–7%/K for both daily and hourly precipitation extremes, about 10% higher than the regional Clausius-Clapeyron scaling of 6.1%/K based on a mean temperature of 24.6 °C. With up to 18% further increase in extreme precipitation under continuing global warming towards the IPCC’s 1.5 °C target, risks of flash floods will exacerbate on top of the current incapability of urban drainage systems in a rapidly urbanizing China.

Global warming is expected to strengthen the hydrological cycle with increased mean precipitation and precipitation extremes^1^,^2^,^3^,^4^,^5^, leading to increasing risks of flash floods^6^,^7^. The moisture holding capacity of the atmosphere grows exponentially with temperature following the Clausius-Clapeyron (C-C) equation that governs saturated vapour pressure, but precipitation may not necessarily do the same^8^,^9^,^10^. Existing observational evidence fails to confirm the expected increase in global land mean precipitation accompanying the overwhelming surface temperature increase^11^,^12^. However, reports have suggested more evident increases in precipitation extremes^5^,^13^,^14^,^15^,^16^.

The increase in moisture supply at a given time in the atmospheric column and surrounding area increases the potential amount of precipitation in a short period of time without reinforcement from lateral transport, leading to amplified precipitation intensity and possibly frequency of occurrence for extreme events^2^. Column integrated water vapour content indicates the total amount of precipitable water, but it never rains out completely. Rainfall intensity does not only depend on local precipitable water, but more importantly on vertical stratification and the lifting force that initiates and maintains the upward motion^2^. Moisture convergence usually plays a very important role to sustain lasting heavy rainfall events that can potentially exceed the C-C limit. A typical example is the heavy rainfall associated with tropical cyclones, in which moisture convergence and the resulting feedbacks through latent heat release are essential.

Limited by available observational measurement, most studies of precipitation extremes are based on daily accumulated total amount^13^,^14^,^15^,^16^,^17^. The indications are that daily precipitation extremes roughly follow the C-C relationship increasing by 6–7% per degree warming, similar to water vapour content. The increase is geographically variable from the high latitudes to the tropics, where daily precipitation extremes actually decrease with rising daily surface air temperatures^17^. However, taking a typical low level winds of 10 m/s, daily precipitation at a specific observational site could potentially be raining out water originating from a radius of over 800 km distance. To examine the true between precipitation intensity and local moisture supply, it is desirable to reduce the temporal resolution of observational measurements or to focus on individual events for the peak intensity. Based on hourly records from specific regions^17^,^18^,^19^,^20^, it is found that extreme precipitation has increased by nearly 14%/K, doubling that from the C-C argument and shown by daily data. Warming induced intensification of precipitation extremes is mainly associated with convective rainfall^19^. Both moisture supply and atmospheric stability may be equally important^20^.

Existing studies use either small number of observational sites or short period of time. Here we analyse, for the first time, hourly precipitation records from 721 observational sites in China over the period 1971–2013 for the summer months June-August. These sites are chosen from over 2400 stations to ensure they have continuous measurements for 43 years with missing data less than 5%.

## Results

**Figure 1:**
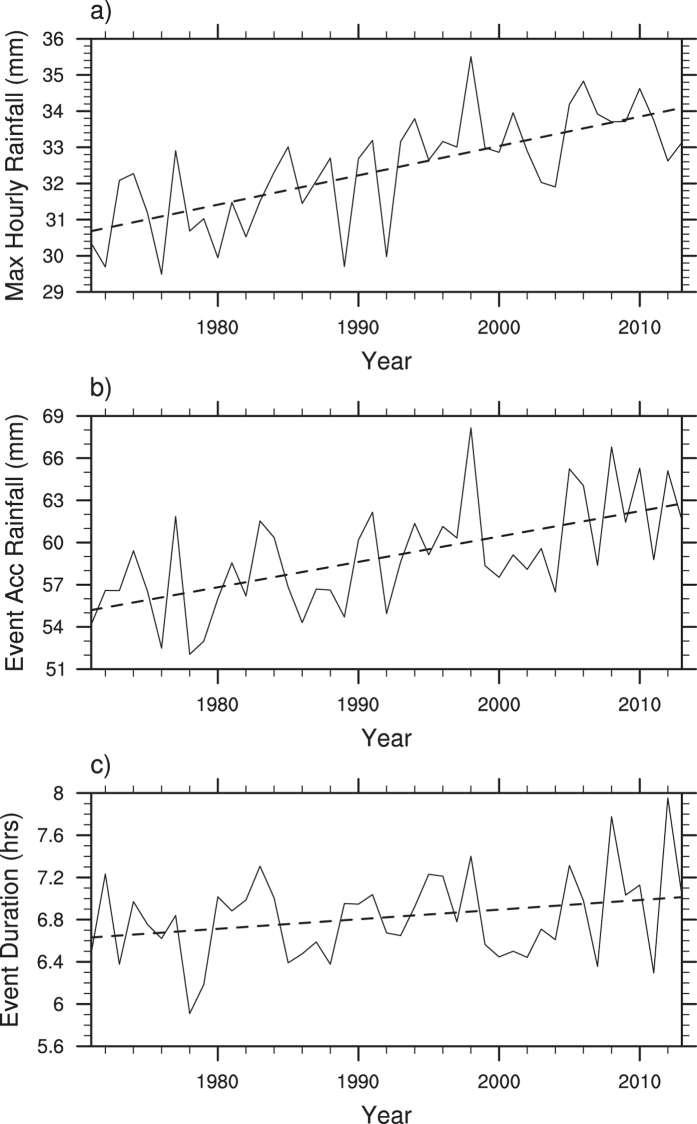
The time series of regionally averaged (**a**) summer maximum hourly precipitation rate (mm/hr), (**b**) the corresponding event accumulated total rainfall (mm) and (**c**) event duration (hrs) using a threshold of 0.1 mm. The dashed lines are the least squared regression lines.

**Figure 2:**
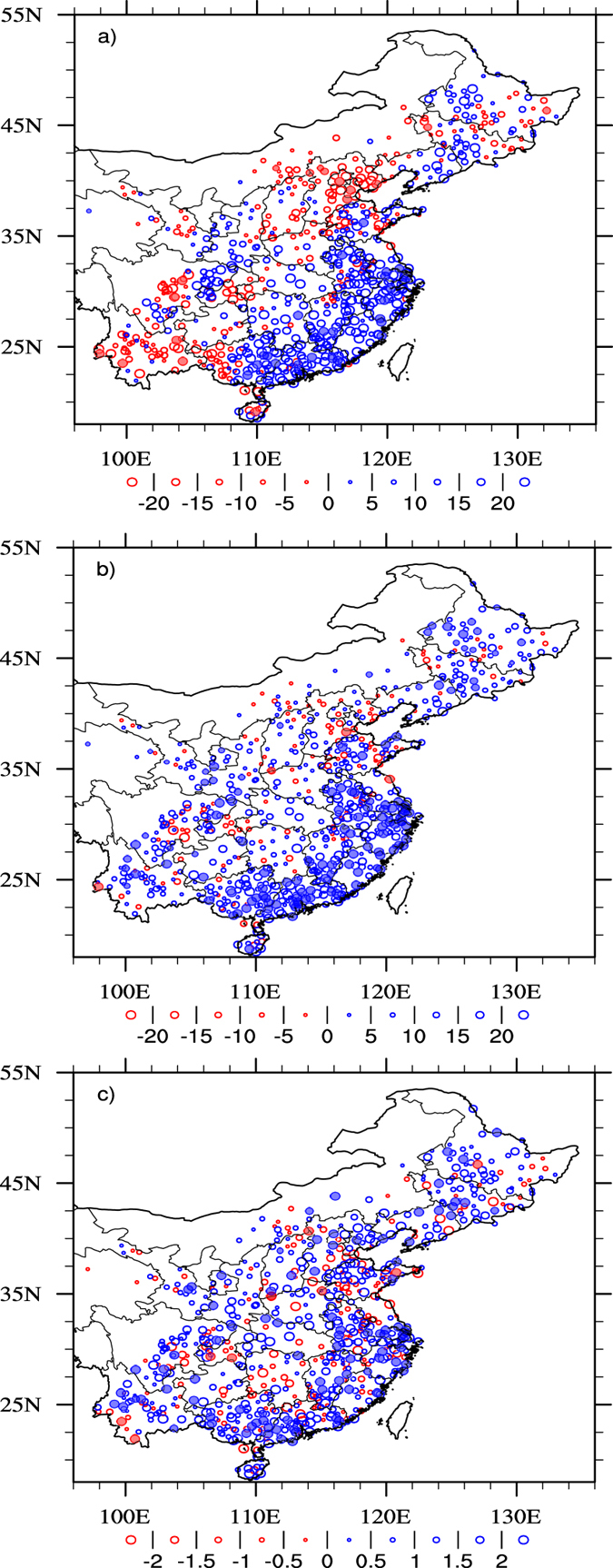
Linear trends (mm/10y) of (**a**) summer total precipitation, (**b**) integral precipitation amount above the 95^th^ percentile of hourly rainfall intensity for wet hours (>0.1 mm/hr), and (**c**) maximum hourly precipitation rate over the period 1971–2013, where solid bullets show that the trends are above the 90% confidence level and the size of the symbol shows the value of the trends with blue being positive. The map was generated using The NCAR Command Language (Version 6.3.0) [Software]. (2016). Boulder, Colorado: UCAR/NCAR/CISL/TDD. http://dx.doi.org/10.5065/D6WD3XH5.

Figure 1a shows the area averaged summer maximum hourly precipitation rate, where the dashed line is the linear fit suggesting an increasing trend of 0.81 ± 0.22 mm/hr for every 10 years at the 90% significance level. The overall increase of hourly extremes is consistent with previous studies^21^,^22^,^23^,^24^. The corresponding event accumulated precipitation amount (Fig. 1b) has seen an upward trend of 1.8 ± 0.6 mm/10y. The event duration (Fig. 1c) has also increased on average by 5.4 ± 4.8 minutes for every 10 years. The distribution of the observational sites can be seen from Fig. 2. The summer total precipitation (Fig. 2a) has shown contrasting patterns with strong upward trends over Southeast China but decreasing trends over North and Southwest China. About 60% of the sites show positive trends while 40% sites show negative trends. The area surrounding Beijing has seen downward trends of up to 7%/10 yr. Precipitation extremes, shown either by the integral precipitation amount above the 95^th^ percentile of hourly precipitation for wet hours (>0.1 mm/hr) in the summer of 1971–2013 (Fig. 2b) or the maximum summer hourly rate (Fig. 2c), have shown a widespread upward trend across China despite negative trends at some stations (19% and 28% respectively). The 95^th^ percentile threshold of 0.1 mm is chosen to ensure sufficient samples for statistical significance. Comparing Fig. 2b and c with Fig. 2a, one finds striking opposite trends between summer total precipitation and precipitation extremes over North and Southwest China. This has particular societal implications because of increasing risks of both drought and flash floods as have been reported somewhere else^25^.

**Figure 3:**
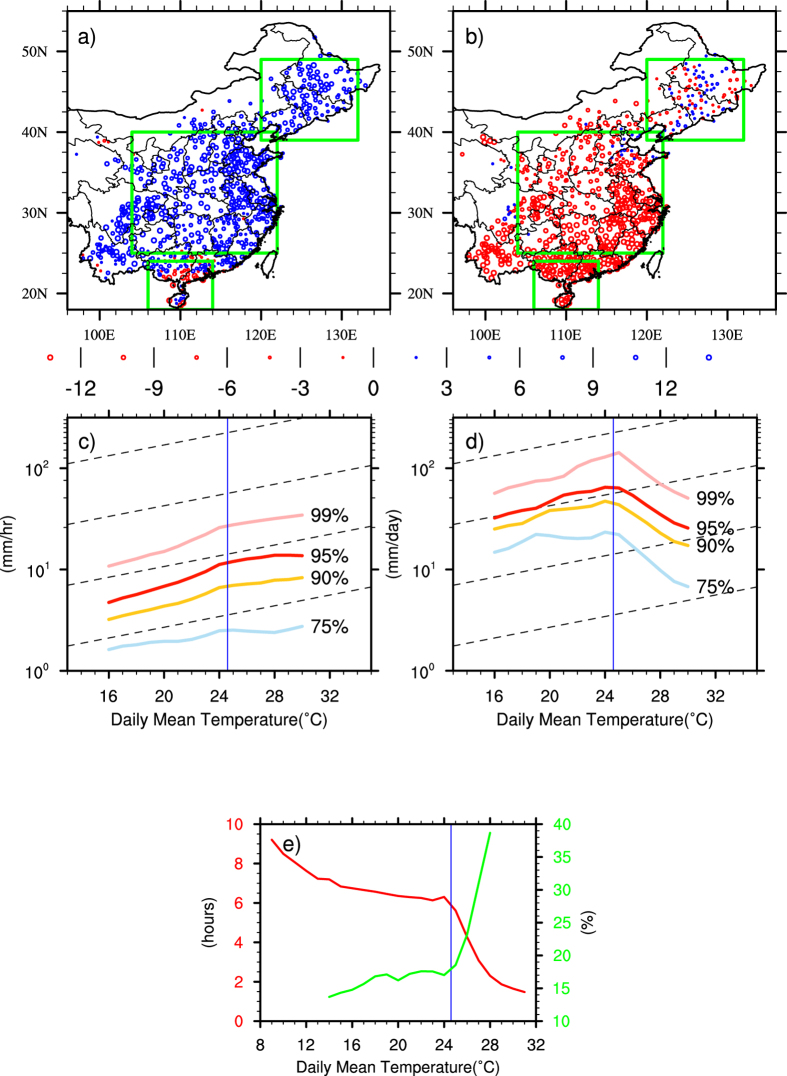
Linear regression coefficients of the 95^th^ percentile of (**a**) hourly (mm/hr/K) and (**b**) daily (mm/day/K) precipitation intensity with daily mean temperature (bin size 1 °C) indicating opposite relationships for an overwhelming majority of observational sites. Scaling rates of the spatially averaged 99^th^, 95^th^, 90^th^ and 75^th^ percentiles of hourly (**c**) and daily (**d**) precipitation intensity with surface daily mean temperature showing temperature dependences with different rainfall intensity categories, where dashed lines indicate the C-C scaling and the blue lines show the regional averaged seasonal mean temperature. (**e**) shows the relationship between daily mean temperature and the number of raining hours on a rainy day (red) in comparison to the relationship between daily mean temperature and the percentage contribution to 95^th^ daily precipitation from the 95^th^ hourly extremes (green). The map was generated using The NCAR Command Language (Version 6.3.0) [Software]. (2016). Boulder, Colorado: UCAR/NCAR/CISL/TDD. http://dx.doi.org/10.5065/D6WD3XH5.

The increase in precipitation extremes is largely attributed to rising temperatures resulting from global warming (e.g. refs 3,16,26). It is known that on monthly mean timescales temperature and precipitation are anti-correlated over land in the mid-low latitudes^27^. The relationship between daily mean temperature and extreme daily precipitation seems to be variable geographically and temperature dependent^17^, although a satisfactory physical explanation remains lacking. Figure 3 shows the linear regression coefficients of the 95^th^ percentile a) hourly and b) daily precipitation intensity with daily mean surface air temperature. Here the percentiles are defined in the same way as in ref. 18. Daily (sums of 24 hourly) and hourly rainfall intensities are both binned into daily mean surface air temperature using 1-degree bin size at each observational site for the entire 43 years. Apart from a small number of observational sites, Fig. 3a and b are dominated by opposite temperature precipitation relationships (over 90%). Hourly precipitation extremes increase with rising temperature, but daily precipitation extremes decrease with higher temperatures. One possible explanation is that hourly precipitation extremes in summer largely occur in the form of convective rainfall mostly in late afternoon to early evenings in hot days^28^. Daily precipitation extremes may occur through longer duration events on wet days. The average durations corresponding to the 95^th^ percentile of daily precipitation extremes are mostly above 10 hours. Actually, less than 50% of extreme hourly precipitation overlaps with daily extremes (i.e. on the same day), particularly over South China (south of 30°N) where less than 40% overlaps. Less than 20% of the daily precipitation extremes reach the threshold of the 95^th^ percentile hourly precipitation extremes. This highlights the difference between daily and hourly precipitation extremes.

As shown by refs 29, 30, 31, 32, the scaling rate of precipitation extremes may be temperature dependent. Figure 3c and d show the spatially averaged temperature dependencies of different rainfall intensity categories, respectively the 99^th^, 95^th^, 90^th^ and 75^th^ percentiles of hourly and daily extremes. The long term summer mean temperature over China is 24.6 °C (marked by the blue line), which corresponds to a C-C scaling of 6.1%/K shown by the dashed lines. Unlike some previous studies, we deliberately avoid the 99.9^th^ percentile because the sample size becomes too small for statistical significance. Agreeing with earlier reports^17^,^18^,^19^,^20^,^29^,^30^,^31^,^32^, it is the most extreme precipitation (presumably convective) that is more sensitive to warmer temperatures. The increase for hourly extremes is monotonic but for daily it is not. Both daily and hourly precipitation extremes show piece-wise linear relationships with surface temperature with a turning point around the seasonal mean summer value of 25 °C. Taking the 95^th^ percentile for example, for temperatures below the mean, both panels show super-C-C scaling of 9.8%/K for hourly and 9.0%/K for daily precipitation. For temperature above the mean, the scaling for hourly precipitation levels off becoming sub-C-C relationship, while for daily extremes, it immediately turns negative with a rate about −18.9%/K. Figure 3e shows the relationship between daily mean temperature and the number of raining hours in a rainy day (red) in comparison to the relationship between daily mean temperature and the percentage contribution to 95^th^ daily precipitation from the 95^th^ hourly extremes (green). The seasonal mean temperature gives a clear separation between the two different measures of precipitation extremes. Below the mean temperature, the two measures represent the same events: presumably large scale precipitation with duration above six hours. The accumulating process, hence moisture convergence, plays a more important role. For temperature above the mean, the two measures are less likely representing the same process, which are mostly intense convective rainfall with short durations around two hours. If they happen to fall into the same category, those hourly heavy downpours dominate the daily measurement. Using the same estimate of 10 m s^−1^ for typical low-level winds, those events are raining out moisture content from an atmospheric column with a radius less than 100 km, which are more likely to be accounted for by the C-C argument. Within Fig. 3a, 28% of the stations show hourly precipitation extremes with daily mean temperature scaling smaller than the cross-country average C-C scaling of 6.1%/K, while 72% show super-C-C scaling with 18% above 2xC-C scaling rate.

**Figure 4:**
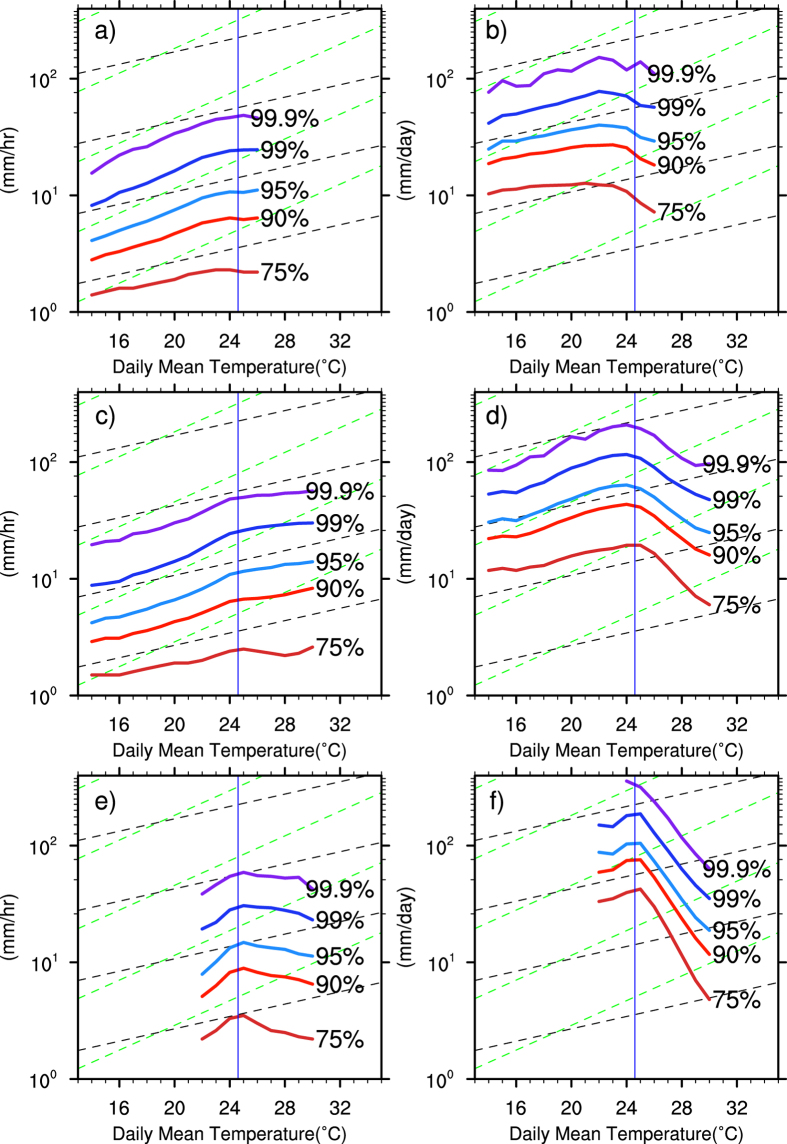
Regional variation of extreme precipitation scaling with daily mean surface air temperature of the 99.9^th^, 99^th^, 95^th^, 90^th^ and 75^th^ percentiles of hourly (left column) and daily (right) for the three selected sub-regions marked in Fig. 3a): North East (**a**,**b**), central China (**c**,**d**), South China (**e**,**f**). For each region, the data was pooled together before statistics are calculated following the example of ref. 30. The dashed lines (black and green) indicate the C-C and double scalings.

As can be seen from Fig. 3a and b, there are regional variations between temperature scalings of daily and hourly precipitation extremes. Figure 4 explores the differences in temperature dependencies between different categories of precipitation extremes and compares their behaviours in daily and hourly measurements for three selected regions with different climate characteristics marked in Fig. 3a by the green boxes. Following ref. 30, data was pooled together for each climate zones before statistics are calculated. Again it is the most extreme precipitation increases the fastest with rising temperature before it reaches the long term seasonal mean. In this way, the scaling rates can double (or even exceeding the doubling rates) of the C-C scaling of 6.1%/K. This is similar to previous report of refs 18,30. In the relatively cold/dry northeast region located over the mid-high latitudes (Fig. 4a,b), both hourly and daily precipitation extremes increase with rising temperature. Daily extremes seem to follow the C-C scaling consistent with many other studies while hourly extremes follow a super-C-C scaling. There is a curving off towards negative scaling for temperature above the seasonal average for daily precipitation extremes while hourly extremes remain positive. For the hot/humid mid-latitude central China (Fig. 4c,d), where the summer climate is largely controlled by the East Asia monsoon and the Meiyu front, both hourly and daily precipitation extremes show a strong super-C-C scaling (12.2%/K) for temperature range roughly between 16 °C and 24.6 °C. For temperature above 24.6 °C, hourly extremes remain positive scaling while daily precipitation extremes show a complete opposite scaling with daily mean temperature. Over South China with tropical climate (Fig. 4e,f), where over 60% summer extreme precipitation comes from tropical cyclones, both hourly and daily precipitation extremes decrease with rising temperature as a result of decreasing number of tropical cyclones^33^. Precipitation extremes only increase for a small temperature range before it reaches the long-term seasonal mean.

**Figure 5:**
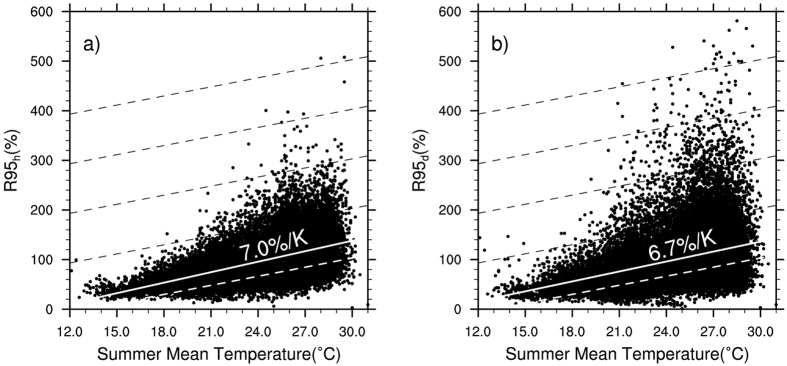
Scatter plots of the summer 95^th^ percentile threshold for hourly (left) and daily (right) precipitation extremes with the corresponding seasonal mean surface air temperatures, showing a consistent supper C-C scaling rate at about 7%/K.

The inconsistency between the scaling rates of precipitation extremes measured by daily and hourly observations with daily mean temperature raises questions about the prevailing popular approaches, in particular the attribution of increasing precipitation to rising temperature under global warming. It also brings in significant uncertainties in future projections of extreme precipitation that have vital implications for climate change impact assessment and adaptation. The physical basis for attributing extreme precipitation to rising temperature is through the indirect C-C argument with moisture holding capacity of the atmosphere. As shown by ref. 10, precipitation rate and precipitation potential or moisture availability are fundamentally different although closely related. Assuming surface air temperature is truly representative of the column integrated moisture content (ignoring the lapse rate), daily precipitation and hourly precipitation may involve very different raining processes with different contributions from horizontal moisture convergence. As pointed out by Refs 2, 20, the moist hydrostatic instability or CAPE (convective available potential energy) plays an important role in precipitation intensity in addition to moisture availability. To determine whether surface air temperature at a given observational site is representative of the large-scale environment, we replace daily mean temperature in our analysis with the seasonal mean. Figure 5 shows the 95^th^ percentile daily (R95_d_) and hourly (R95_h_) precipitation extremes relationships with seasonal mean surface air temperature for all the 721 stations. With obvious spread due to regionalities, the coherent relationship is clear and robust. Moreover, we now see a good agreement between the scaling rates of daily precipitation extremes with that of hourly.

## Discussion

This study has analysed a large dataset of continuous precipitation records with high spatial (721 weather stations) and temporal resolution (hourly) covering a period of 43 years from 1971 to 2013. This is probably so far the most comprehensive study on summer precipitation extremes employing over 68 million observations. A robust conclusion is that the regionally averaged maximum summer hourly rainfall rate across a large part of China has increased by 11.2%. Hourly and daily precipitation records can lead to very different conclusions, particularly for their temperature dependencies. A neutral point which is the local seasonal mean temperature provides the best environmental climate background linked to moisture supply, for which both daily and hourly extreme precipitation intensities scale with around the C-C rate. Beyond the summer seasonal mean (until 30 °C), hourly precipitation extremes continue to intensify at a sub-C-C rate while daily precipitation extremes decrease rapidly with rising temperatures. We have inferred that it is probably the extreme convective summer rainfall continues to intensify, but further investigation such as refs 19,20,34 are urgently needed to confirm that.

An intriguing phenomenon is that the positive correlation between precipitation extremes and surface air temperature is only found within certain intermediate temperature range, beyond which the relationship reverses. This is particularly true with daily extremes and has also been reported in previous studies such as refs 17,31 and others. We have in this paper, for the first time, offered an explanation of this phenomenon; again highlighting the fundamental point that precipitation is not a monotonic function of temperature even though the atmosphere’s moisture holding capacity is^10^. Moisture availability alone cannot explain the rate of intensification of precipitation extremes. Other dynamic and thermodynamic factors, such as CAPE and the dynamic effects of latent heat release associated with extreme precipitation.

Urban flooding has seen acceleration over China in recent decades due to rapid urbanization and incapability of outdated drainage systems^7^, which has been aggravated by increasing extreme precipitation accompanying a rapidly warming climate. A survey by the residential development department in China has found that 62% of cities were flooded at least once between 2008 and 2010, based on a sample of 351 Chinese cities (http://news.ifeng.com/shendu/nfzm/detail_2013_07/18/27643337_0.shtml). During the period 1971–2013, the regional average summer mean temperature across a large part of China has increased by ~0.95 °C, smaller than the annual mean rise of 1.44 °C^34^, but the regionally averaged maximum summer hourly precipitation has increased by 11.2%. Using various different measures, we have shown that hourly and daily summer precipitation extremes in China increase robustly at super-C-C scalings with rising temperatures. Continuing global warming is to further strengthen the hydrological cycle with increased mean precipitation and precipitation extremes^3^,^4^,^5^,^35^. If the current trend continues, further increases in extreme precipitation and flooding risks are expected. Even if the 1.5 °C IPCC target is achievable, one expects a further intensification of precipitation extremes in China by at least another 10%. In some regions, that increase can be as large as 18%. Without forward-looking measures, the implied economic damage can be huge^36^.

## Data and Methods

Data used in this study was provided by National Meteorological Information Center, China Meteorological Administration, including quality controlled daily mean surface air temperature and precipitation^37^, as well as hourly precipitation rate^38^,^39^. 721 weather stations were selected from over 2400 observational sites for the summer month June, July, and August over the period 1971–2013 to ensure missing data at each site are no more than 5%.

A wet event is defined as such that the precipitation rate is larger than 0.1 mm for both daily and hourly observations. Detailed methods are provided in figure captions for each individual plot. Standard percentile technique is applied for precipitation intensity categories. When calculating precipitation temperature dependencies, a 1° bin size is used for temperature. Figure 4 was produced by pooling data from the specific climate zone together before statistics are calculated.

## Additional Information

**How to cite this article**: Xiao, C. *et al*. Robust increase in extreme summer rainfall intensity during the past four decades observed in China. *Sci. Rep.* **6**, 38506; doi: 10.1038/srep38506 (2016).

**Publisher's note:** Springer Nature remains neutral with regard to jurisdictional claims in published maps and institutional affiliations.
